# Prevalence and associated risk factors of visual impairment in school children: mHealth-based vision screening in government schools of Rawalpindi, Pakistan

**DOI:** 10.3389/fmed.2025.1661710

**Published:** 2025-10-21

**Authors:** Ayesha Javed, Shamaila Mohsin, Ramsha Habib, Uzma Ahsan Malik, Abdul Momin Rizwan Ahmad

**Affiliations:** ^1^National University of Medical Sciences (NUMS), Rawalpindi, Pakistan; ^2^Department of Public Health, Armed Forces Postgraduate Medical Institute (AFPGMI), National University of Medical Sciences (NUMS), Rawalpindi, Pakistan; ^3^Department of Pediatric Medicine, Hameed Latif Hospital, Lahore, Pakistan; ^4^Department of Health Sciences, University of York, York, United Kingdom; ^5^Department of Human Nutrition and Dietetics, NUST School of Health Sciences, National University of Sciences & Technology (NUST), Islamabad, Pakistan

**Keywords:** visual impairment, preventable blindness, school age children, mHealth-based vision screening, Pakistan, risk factors

## Abstract

**Background:**

School-based vision screening plays a vital role in identifying visual problems at an early stage. There is limited evidence on the frequency of visual impairment and its associated risk factors among school-aged children.

**Objective:**

This research aimed to assess the prevalence of visual impairment and its associated risk factors among school children enrolled in Government Schools of Rawalpindi.

**Methodology:**

An analytical cross-sectional study was conducted among 320 schoolchildren in the Rawalpindi district of Punjab province from July to December 2024. Presenting visual acuity was assessed using the validated Peek Acuity app on smartphone with the tumbling E optotypes. Visual impairment was defined as presenting visual acuity worse than 6/12, based on failure to correctly identify at least four out of five optotypes at the 6/12 level at a testing distance of 3 m. A validated questionnaire was utilized to assess risk factors associated with visual impairment. The prevalence of visual impairment was presented as frequencies and percentages. Binary logistic regression was performed with visual impairment as the dependent variable, considering age, gender, father's educational status, mother's educational status, household income, maternal illness during pregnancy, parent with visual impairment, sibling with visual impairment, birth weight, gestational age, complications at birth, serious infection during childhood, history of head trauma or injury, duration of television exposure, and duration of mobile/computer exposure as independent variables. Variables with a *p* < 0.05 and a 95% confidence interval in the multivariate model were considered statistically significant.

**Results:**

A total of 320 study participants were included in this study. Visual impairment was identified in 82 children (25.6%), consisting of 35 males (42.68%) and 47 females (57.3%). It was categorized as mild in 30 (36.58%) and moderate in 52 (63.41%) children. In multivariate analysis, parent with visual impairment [4.201 (2.221–7.948)], low birth weight [0.376 (0.189–0.749)], small gestational age [0.231 (0.113–0.475)] and exposure to mobile and computer devices [2.368 (1.040–5.393)] were factors significantly associated with visual impairment.

**Conclusion:**

This study identified a high burden of visual impairment among schoolchildren, with a greater proportion observed in females and predominantly presenting with moderate severity.

## 1 Introduction

According to universal estimates, there are around 19 million children with visual impairment globally, the majority of whom live in low- and middle-income countries (LMICs) (1). Pakistan ranks third among 20 countries most affected by blindness and moderate to severe visual impairment, following India and Bangladesh (2). It is estimated that over two million children in Pakistan are living with blindness or visual impairment (3). However, the burden of vision loss in Pakistan in the last one decade remained unclear (2).

Visual impairment in children is caused by a variety of factors, with uncorrected refractive errors being the leading cause, both in children and adults (4). Additionally, recent systematic review (2000–2020) reported that uncorrected refractive errors are the most prevalent cause of vision impairment among children (1).

Evidence indicates that uncorrected refractive errors in infancy and early childhood may lead to developmental delays (5) and are associated with clinically recognized deficits in cognitive and visual-motor skills (6). Visual impairment during childhood poses significant barriers to learning (7), development, and academic achievement, which can ultimately affect future employment prospects and socioeconomic standing (8, 9).

The World Health Organization (WHO) has prioritized the correction of refractive errors as one of the target areas to eliminate avoidable causes of visual impairment (10, 11) while majority of causes of visual impairment can be prevented or treated using highly cost effective measures (12). Conventional ophthalmic tools are expensive, non-portable, and require specialized training, limiting their accessibility (13). Thus, there is a need for accessible, self-manageable, and automated tools for visual acuity tests to increase early detection and timely assistance for those with visual impairment (14). With rising global smartphone use, mobile health (mHealth) approaches are increasingly recognized as cost-effective, user-friendly tools for early detection and management of visual impairment (15). Some studies have introduced innovative mobile-based visual acuity testing methods, including a validated mobile app shown to produce results comparable to standard Snellen charts (16, 17).

This study investigates the use of smartphone-based vision screening to address the high burden of undiagnosed visual impairment among schoolchildren in underserved communities of Rawalpindi, Pakistan. In addition, this study aimed to identify the underlying factors associated with visual impairment among children, addressing a critical gap in the existing evidence.

## 2 Materials and methods

**Figure 1:**
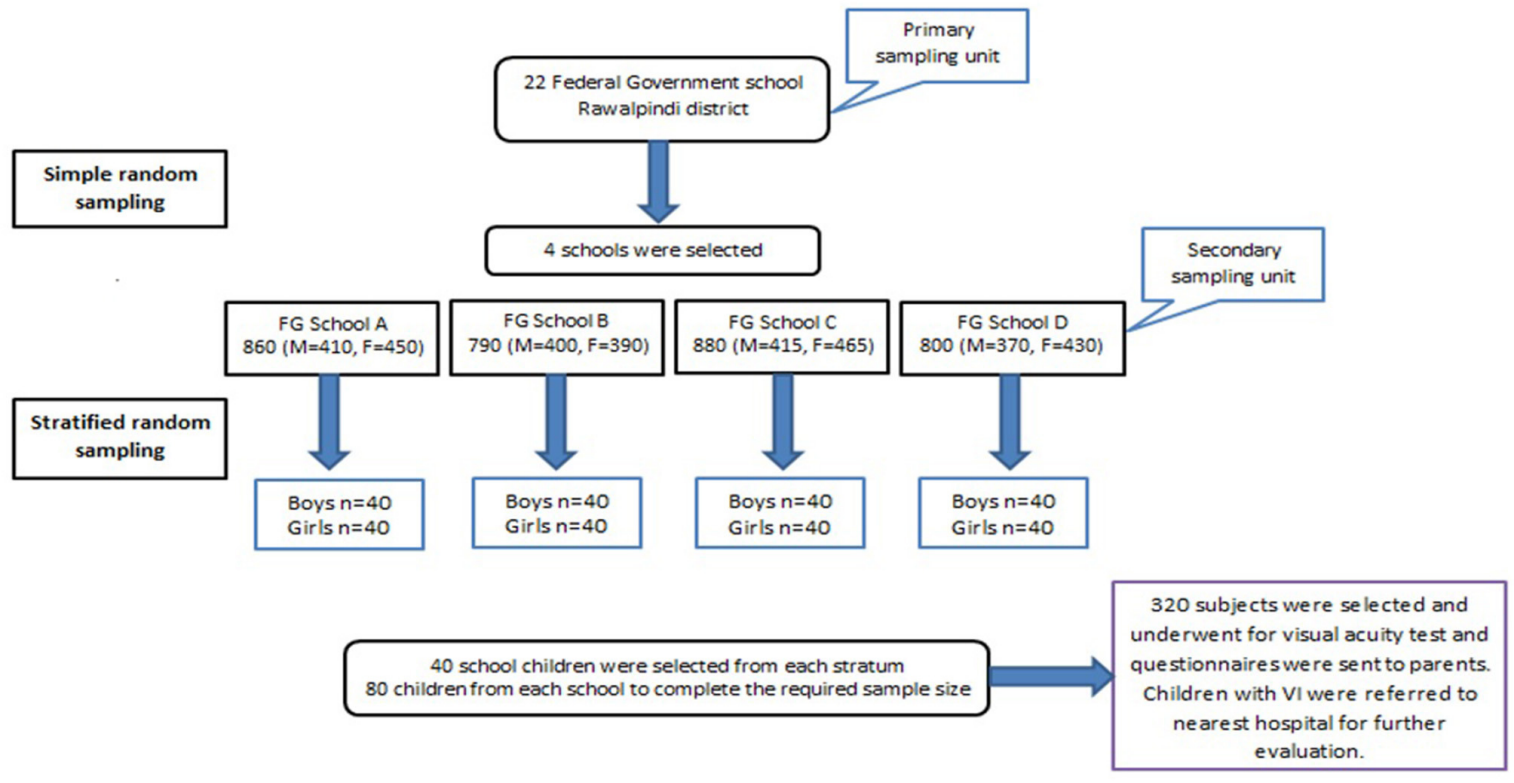
Schematic representation of sampling techniques in Federal Government Schools, Rawalpindi.

An analytical cross-sectional study was conducted in Federal Government schools in Rawalpindi district of Punjab province from July to December 2024 using two stage probability sampling technique. Based upon the list of all FG schools obtained from FGEI, four schools among 22 were selected randomly using computer generated numbers. The total sample size was distributed proportionally based on their population size to the selected schools. Next the stratified random sampling was employed. A list of students was obtained from each school. Children were stratified on the basis of age and gender and selected proportionally (Figure 1). Study participants were 5–15 years of age as per WHO recommendations for vision screening (18), male and female (in equal proportion).

Using the Cochrane formula (https://dissertationdataanalysishelp.com/cochrans-sample-size-calculator/), a sample size of 272 was calculated with a 95% confidence interval, 5% margin of error, and 21.8% population proportion (19). After adjusting for a 20% non-response rate, the final sample size was 320. Pupils aged 5–15 years, with parents' written informed consent submitted timely to the teachers were eligible for the participation. Children with known refractive error or using spectacles or with an apparent eye disease (such as conjunctivitis, red eye, ocular trauma, trachoma, etc.) were excluded.

A meeting was held with the principal of each FG school to explain the research objectives. A brief awareness session was conducted during the school assembly to inform students and parents about the importance of vision screening, followed by an interactive Q&A session. Key study details were communicated to caregivers and participants through school staff. All data was kept confidential and private, using coding and aggregate reporting to ensure anonymity by removing identifiable information. The data was used solely for research purposes and was not shared with any third party maintaining strict confidentiality.

External eye diseases were identified by external eye examination with the help of a medical torch. Children who met the inclusion criteria underwent the visual acuity test using the validated Peek Acuity app (https://peekvision.org/solutions/peek-acuity/). This app, which includes a comprehensive software platform with data collection capabilities, was used on a smartphone. The Peek Acuity app has demonstrated a sensitivity of 84.6%, specificity of 97.7%, positive predictive value of 68.8%, and negative predictive value of 99.1% (17, 20). It employs a smartphone-based tumbling-E optotype displayed in four orientations, the participant indicates the direction, and the tester inputs the response via swipe. The test uses a single isolated optotype without crowding bars, presented one at a time in random orientations, thereby minimizing the tester bias. All testing was performed using a Samsung Galaxy (Tab A 10.1-inch) tablet, with manual calibration performed prior to testing to ensure optotypes were displayed at the correct physical size. In accordance with Peek Acuity's calibration guidance for this screen size, the test distance was fixed at 3 m under natural daylight room illumination. Results are output in both LogMAR and Snellen (metric/imperial), with a visual blur simulation to aid participant understanding.

**Figure 2:**
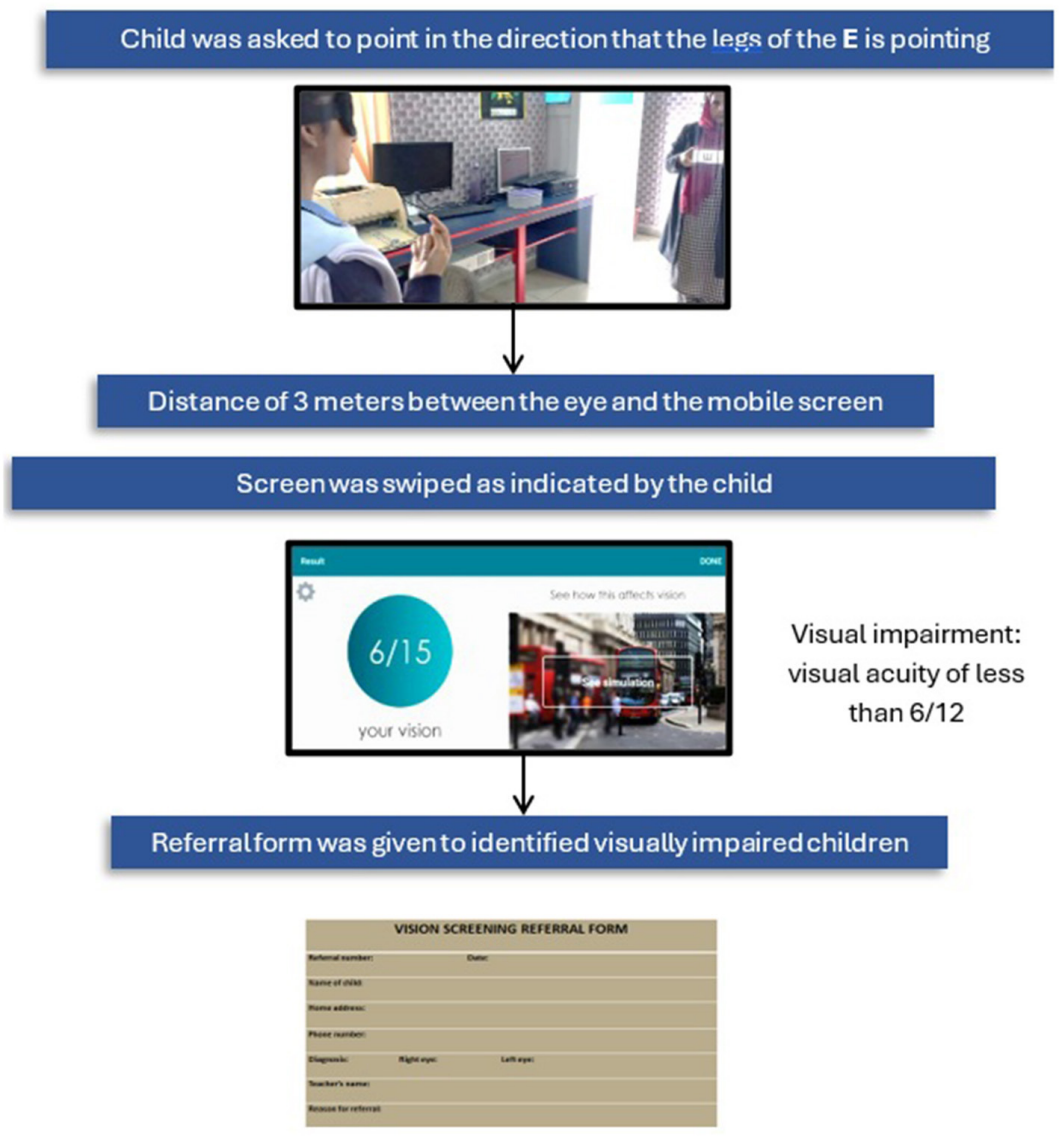
Vision screening procedure.

The right eye was tested first, followed by the left eye. Visual impairment was defined as presenting visual acuity worse than 6/12 in the better eye, determined by correctly identifying four out of five optotypes at the 6/12 level, when tested at a distance of 3 m under natural daylight room illumination (Figure 2). According to International Classification of Diseases 11th revision, 2018, following cut-off values for the severity of visual impairment were used (21). Mild –presenting visual acuity worse than 6/12 but better than 6/18 in the best-corrected eye. Moderate- presenting visual acuity worse than 6/18 but better than 6/60. Severe- presenting visual acuity worse than 6/60 but better than 3/60. Blindness as VA worse than 3/60 in the better eye with the best possible correction.

Participants with VA worse than 6/12 in “either eye” were re-examined and those who continued to meet the criteria for visual impairment were given a referral slip to the nearest hospital for further evaluation. An Urdu version of the validated and structured closed-ended questionnaire (22) were used to assess risk factors associated with visual impairment, which were prepared following research and consultation with experienced experts in the subject area (see Supplementary material). The questionnaire was pre-tested in pilot study before data collection.

Data were entered and analyzed using SPSS version 27 software and checked for normality by applying Kolmogorov-Smirnov (KS) test. Sociodemographic characteristics of participants were presented in tables using simple frequency distribution and percentages for categorical data.

Percentage used to present the frequency of visual impairment among study participants. Potential risk factors associated with visual impairment were first analyzed by comparing children with and without visual impairment in bivariate models. If significant differences will be shown in the univariate model, these risk factors will be included together in multiple logistic regression models with visual impairment as dependent variable. Variables with *p* < 0.05 and 95% confidence interval in the multivariate model were considered statistically significant. To overcome the non-response rate, adjusted sample size with 20% non-response rate used.

## 3 Results

**Figure 3:**
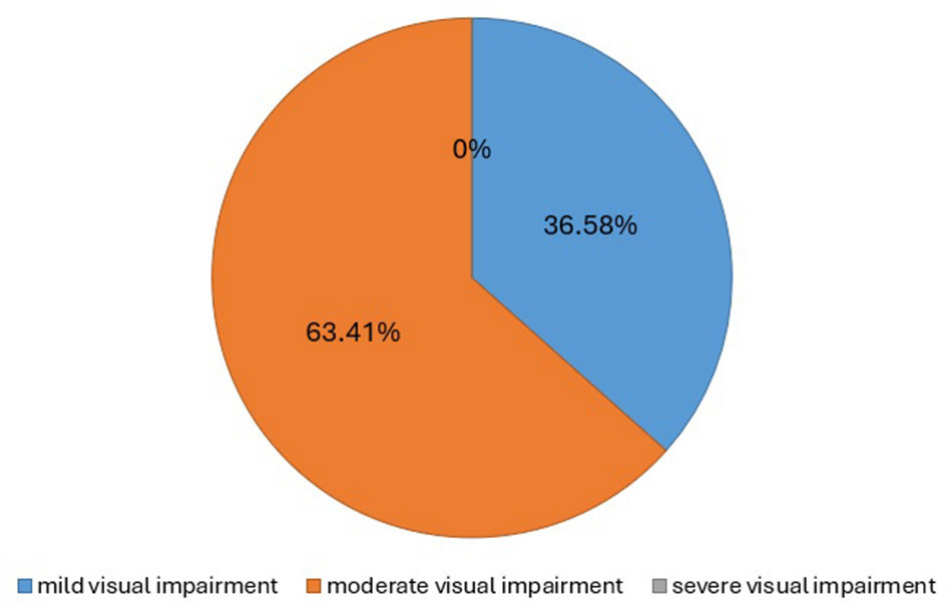
Severity of visual impairment.

A total of 320 children participated in this study, all of whom were aged between five and fifteen years and were evaluated for visual impairment. The baseline characteristics and frequency of risk factors among participants were assessed (Tables 1, 2). Visual impairment was identified in 82 children (25.6%), consisting of 35 males (42.68%) and 47 females (57.3%). Among these, 38 children (46.3%) were aged 5–10 years, while 44 children (56.7%) were aged 11–15 years (Table 3). The severity of visual impairment was categorized as mild in 30 (36.58%) children and moderate in 52 (63.41%) children (Figure 3).

**Table 1:** Sociodemographic characteristics of participants.

**Variables**	**Frequency**	**Percentage (%)**
**Age (years)**		
5–10	160	50
11–15	160	50
**Gender**		
Male	160	50
Female	160	50
**Father educational status**		
None	25	7.8
Primary	109	34
Secondary	186	58
**Mother educational status**		
None	33	10.3
Primary	116	36.2
Secondary	171	53.4
**Household income**		
Low-less than PKR 42,000	141	44
Lower middle-PKR 42,000–99,999	150	46.8
Middle-PKR 1,00,000–1,49,000	29	9

**Table 2:** Frequency of risk factors among participants.

**Variables**	**Frequency**	**Percentage (%)**
**Maternal illness during pregnancy**		
Yes	67	21
No	253	79
**Parent with visual impairment**		
Yes	108	33.7
No	253	66.2
**Sibling with visual impairment**		
Yes	59	18.4
No	261	81.5
**Birth weight (BW) (g)**		
Low BW < 2,500 g	54	16.8
BW 2,500 g or >2,500 g	266	83.1
**Gestational period (weeks)**		
GA < 37weeks	89	27.8
GA 37 weeks or >37 weeks	231	72.2
**Complications at birth**		
Yes	30	9.3
No	281	87.8
**Serious infections during childhood**		
Yes	53	16.5
No	267	83.4
**Head trauma or eye injury**		
Yes	15	4.7
No	305	95.3
**Duration of TV exposure**		
< 2 h	71	22.1
2–4 h	146	45.6
>4 h	103	32.1
**Duration of mobile/computer exposure**		
< 2 h	85	26.6
2–4 h	123	38.4
>4 h	112	35

**Table 3:** Frequency of visual impairment by age and gender.

**Age (years)**	**Visual impairment**				**Total**
	**Male**		**Female**		
	**Frequency**	**Percentage %**	**Frequency**	**Percentage %**	
5–10 years	12	14.63	26	31.7	38 (46.3%)
11–15 years	23	28.04	21	25.6	44 (56.7%)
Total	35 (42.68%)		47 (57.31%)		82 (25.6%)

A logistic regression model was employed to identify risk factors influencing visual impairment in children. The univariate analysis revealed a significant odds ratio (OR) with a 95% confidence interval (CI) and a *p*-value of < 0.05. The univariate (crude) logistic regression results for all independent variables are presented in Table 4. It was found that children with a positive family history of VI among parents exhibited a significantly higher prevalence of vision impairment [COR 4.849 (2.845–8.266)]. Additionally, low birth weight [COR 0.083 (0.42–0.161)], small gestational age [COR 0.147 (0.085–0.256)] and complications at birth among children [COR 2.360 (1.277–4.363)] was linked to a notably increased prevalence. Similarly, a significant association was found among those exposed to mobile devices or computers for more than 4 h [COR 2.216 (1.25–3.926)].

**Table 4:** Univariate (crude) and multivariate logistic regression of factors associated with visual impairment among schoolchildren.

**Variable**	**Visual impairment**		**Univariate analysis COR (95% CI)**	**Multivariate analysis AOR (95% CI)**
	**Yes (*****n*** = **82)**	**No (*****n*** = **238)**		
**Age (years)**				
5–10	38	122	1.218 (0.736–2.014)	
11–15	44	116		
**Gender**				
Male	35	125	1.485 (0.895–2.464)	
Female	47	113		
**Father educational status**				
None	09	16	1	
Primary	34	75	0.472 (0.194–1.148)	
Secondary	39	147	0.585 (0.342–1.002)	
**Mother educational status**				
None	10	23	1	
Primary	35	81	0.635 (0.278–1.452)	
Secondary	37	134	0.639 (0.373–1.095)	
**Household income**				
Low-less than PKR 42,000	47	94	1	
Lower middle-PKR 42,000–99,999	30	120	0.417 (0.149–1.161)	
Middle-PKR100,000–149,000	5	24	0.833 (0.294–2.365)	
**Maternal illness during pregnancy**				
Yes	19	48	1.194 (0.653–2.181)	
No	63	190		
**Parent with visual impairment**				
Yes	50	58	4.849 (2.845–8.266)	4.201(2.221–7.948)^***^
No	32	180		
**Sibling with visual impairment**				
Yes	19	40	1.493 (0.807–2.762)	
No	63	198		
**Birth weight (BW) (g)**				
Low BW < 2,500g	22	32	0.155 (0.089–0.268)	0.376 (0.189–0.749)^**^
BW 2,500 g or >2,500 g	60	206		
**Gestational period (weeks)**				
GA < 37weeks	48	41	0.147 (0.085–0.256)	0.231 (0.113–0.475)^***^
GA 37 weeks or >37 weeks	34	197		
**Complications at birth**				
Yes	30	9	2.360 (1.277–4.363)	1.293 (0.563–2.970)
No	36	245		
**Serious infections during childhood (trachoma, measles)**				
Yes	16	37		
No	66	201	1.317 (0.688–2.520)	
**Head trauma or eye injury**				
Yes	6	9	2.009 (0.692–5.828)	
No	76	229		
**Duration of TV exposure**				
< 2 h	10	61	1	
2–4 h	45	101	1.402 (0.627–3.13)	
>4 h	27	76	2.167 (0.974–4.823)	
**Duration of mobile/computer exposure**				
< 2 h	12	73	1	
2–4 h	27	96	3.791 (1.848–7.785)	2.368 (1.040–5.393)^*^
>4 h	43	69	2.216 (1.25–3.926)	

^*^p < 0.05; ^**^p ≤ 0.01; ^***^p ≤ 0.001.

COR, crude odds ratio; AOR, adjusted odds ratio.

(All independent variables tested in the univariate analysis are presented, regardless of statistical significance).

**Figure 4:**
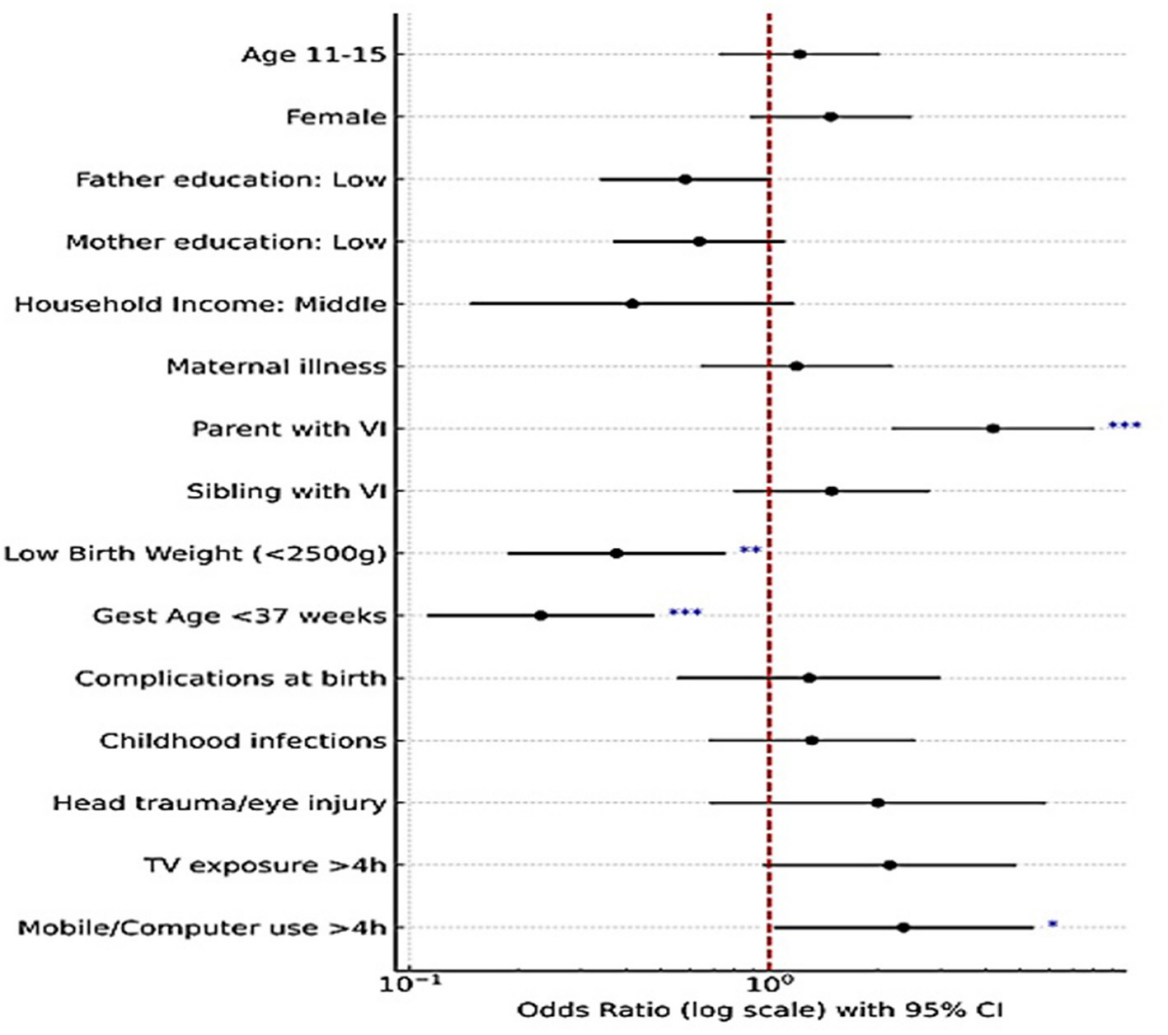
Forest plot of factors associated with visual impairment (results of bivariate and multivariate logistic regression; with 95% CI; significance indicated as *p < 0.05; **p ≤ 0.01; ***p ≤ 0.001).

Variables significant at the univariate level were subsequently included in the multivariate logistic regression model. In the adjusted analysis, four variables remained independently associated with visual impairment: parental visual impairment [AOR 4.201 (2.221–7.948)], low birth weight [AOR 0.376 (0.189–0.749)], small gestational age [AOR 0.231 (0.113–0.475)] and prolong exposure to mobile and computer devices [AOR 2.368 (1.040–5.393)]. The pooled results are presented in Figure 4, which shows the forest plot summarizing the overall effect estimates.

Furthermore, to check for potential multicollinearity among independent variables, variance inflation factors (VIFs) and tolerance values were examined. All variables included in the final model demonstrated acceptable VIF values (< 3) and tolerance >0.3 (23), indicating no significant collinearity and ensuring the stability of the regression estimates.

## 4 Discussion

This study contributes important data on childhood vision health in low-to-middle-income communities (LMICs) by reporting the frequency of visual impairment among school children, in contrast to previous regional studies. mHealth-based screening tools represent an emerging innovation with substantial potential to enhance health outcomes. Our study demonstrated significant improvements in early detection, particularly in resource-limited settings, where such tools prove especially effective. The result shows a notable prevalence of visual impairment among school children compared to previous regional studies. A study conducted in 2018 reported a prevalence of 17.5% (24), while another study in 2016 found a prevalence of 12.4% (25). The 2017 national survey in Pakistan reported a prevalence of 15.38%, which projected a rise in the coming years (4). International studies, including those from South Africa (26) and Egypt (27), have reported varying prevalence rates ranging from 16% to 29.4% further highlighting the variability of VI rates across different regions and populations.

The disparity could be attributed to a variety of factors, including differences in study design, the target population, and the specific inclusion criteria used. Economic disparities, ethnic differences, and access to healthcare services likely play a significant role in influencing the prevalence of visual impairments (28). Additionally, varied operational definitions taken into consideration by investigators are the reason for variations in prevalence data from research conducted in other Pakistani cities and even in other countries (29).

Family history emerged as a strong risk factor for visual impairment in our study. This association is a common finding across multiple studies (23, 26, 30). A family history of myopia, in particular, has been linked to a higher risk of visual impairment as mentioned in a review that focused on the prevalence and etiology of VI (31). Genetic contributions to visual impairment have been demonstrated through familial and genome-wide association studies, indicating that its etiology is complex (32).

We found that among the birth-related factors, low birth weight (< 2,500 g) was a significant risk factor for visual impairment. Children with low birth weight were more likely to experience VI, with an OR of 2.45 (95% CI, 1.14–5.26) (33). Low birthweight was also identified in other comparable studies that have demonstrated the association between low birth weight and an increased risk of refractive errors and other visual disorders (34, 35). Studies have shown that low birth weight disrupts the process by which the eye becomes more focused as a child grows, leading to a higher incidence of refractive errors (36).

Another important finding in our study was the increased risk of visual impairment among preterm children, who are more likely to experience ocular disorders such as refractive errors. This finding corroborates study from Ethiopia in 2024 which reported refractive errors were the leading type of ocular morbidity seen in 115/222 (51.8%) preterm children (36). Similarly, a report from Sweden (37) and Africa (38), found that nearly half of the children who were screened for preterm had refractive error. The association between prematurity and visual impairment may be linked to the underdevelopment of the visual system, which is more vulnerable in preterm children (39).

Lastly, our study also highlights the high prevalence of visual impairment among students involved in near work activities, including screen time (video games, computers, and mobile devices). This aligns with findings from studies conducted in other regions in which the children who spent more than 4 h per day engaged in near work activities were found to have a significantly higher prevalence of visual impairment (28). Prolonged near work activities have been shown to increase the risk of developing refractive errors, particularly myopia, as the eye undergoes excessive accommodation during close-up tasks (40).

This study addresses an important area with limited reviews in the existing literature and has several strengths including use of a two-stage sampling design, use of validated tool, appropriate sample size and finally analysis through robust analytical approach to assess the prevalence and associated risk factors. However, there are several limitations. The study utilized basic screening tests for visual impairment instead of comprehensive clinical eye examinations. While these screening tests can detect obvious visual impairments, they may overlook more subtle cases of refractive errors or other ocular conditions. In addition, visual impairment classification depends on uncorrected visual acuity (UCVA) instead of best-corrected visual acuity (BCVA). The study also employed a single optotype acuity task rather than full line-based acuity testing, which may overestimate visual performance and limit comparability with studies using standard line acuity measures. As this work was limited to school-based screening, the subsequent referral process and outcomes were not captured. Consequently, information on the proportion of referred children who received further evaluation and the specific types of refractive errors identified could not be determined. Longitudinal studies would be better suited for tracking the progression of visual impairment over time and for examining the causal relationships between risk factors and visual impairment. Our research concentrated on children from low-income communities, which may restrict the generalizability of our findings to other regions or socio-economic groups. Moreover, environmental factors such as air pollution and nutritional deficiencies, which could significantly impact the development of visual impairment, were not considered in this study.

## 5 Conclusion

This study identified a high burden of visual impairment among schoolchildren, with a greater proportion observed in females and predominantly presenting with moderate severity. Multivariate analysis revealed that parental history of visual impairment, exposure to mobile and computer devices, low birth weight, and small gestational age were significantly associated factors. Future research should evaluate referral uptake and the spectrum of refractive errors detected to strengthen the evidence for integrated screening-to-care pathways. Additional studies in other regions of Pakistan are required to obtain a more comprehensive understanding of the national burden of visual impairment among children.

## Data Availability

The raw data supporting the conclusions of this article will be made available by the authors, without undue reservation.
