# Supplementary material for: Prevalence and associated risk factors of visual impairment in school children: mHealth-based vision screening in government schools of Rawalpindi, Pakistan
Source: Front Med (Lausanne). 2025 Oct 21;12:1661710. doi: 10.3389/fmed.2025.1661710 (PMC12582945; doi:10.3389/fmed.2025.1661710)
Supplement: Supplementary file 1 [file Supplementary_file_1.pdf]

PARENT QUESTIONNAIRE

Risk assessment tool for visual impairment (A. Chia et al., 2013)

Consent statement:

☐ I understand that all information I provide for this study will be treated confidentially

☐ I voluntarily agree to participate in this research study: Prevalence and associated risk factors of visual impairment in school children: mhealth-based vision screening in government schools of Rawalpindi, Pakistan

**Background information**		
Name of school:	Grade:	
Name of student:	DOB:	
Age:	Gender:	
**BIRTH FACTORS**		
How much did your child weight at birth?	Birth weight ≤ 2500gms ☐<br>Birth weight ≥ 2500gms ☐	
How was your child's gestational age at birth?	GA ≤ 37 weeks ☐<br>GA ≥ 37 weeks ☐	
**FAMILY HISTORY**		
What is your total combined monthly household income?	less than 42,000 PKR ☐<br>42,000 PKR – 99,999 PKR ☐<br>100,000 PKR – 149,999 PKR ☐<br>Above 150,000 PKR ☐	
What's the child father's completed educational level?	None ☐<br>Primary ☐<br>Secondary ☐	
What's the child mother's completed educational level?	None ☐<br>Primary ☐<br>Secondary ☐	
Parent with visual impairment	**YES**	**NO**
Sibling with visual impairment	**YES**	**NO**

MATERNAL RISK FACTORS		
Maternal age at birth of child	17 years or below<br>20 years to 35 years<br>Above 35 years	
Marital status	Married<br>Widowed<br>Divorced	
Maternal illness during pregnancy	Diabetes/Hypertension<br>Any other	
**POSTNATAL RISK FACTORS**		
Were there complications immediately after birth or admission to NICU	**Yes**	**No**
Since birth, has the baby had any serious infections such as trachoma, measles	**Yes**	**No**
Since birth, has the child suffered significant head trauma or eye injury	**Yes**	**No**
Duration of TV exposure<br>< 2 hours<br>2-6 Hours<br>> 4 hours		
Duration of mobile/computer exposure<br>< 2 hours<br>2-6 hours<br>> 4 hours		
